# Experience with injectables performed at a resident department Aesthetic Surgery Clinic

**DOI:** 10.1016/j.jpra.2026.01.015

**Published:** 2026-01-16

**Authors:** Carter J. Boyd, Hani Y. Nasr, Michael F. Cassidy, Zachary M. Borab, Neil M. Vranis, Alexis K. Gursky, Rebecca Gober, Barry M. Zide, Daniel J. Ceradini

**Affiliations:** Hansjörg Wyss Department of Plastic Surgery, NYU Langone Health, New York, NY, USA

**Keywords:** Injectables, Minimally-invasive aesthetic surgery, Resident aesthetic education, Facial aesthetics

## Abstract

**Background:**

The popularity and rapid growth of aesthetic injectables, in conjunction with the influx of competing specialties performing these procedures, underscores the importance of competence of plastic surgery residents and rigorous modalities of plastic surgery training.

**Objectives:**

Our objective was to review injectables and outcomes from our plastic surgery resident aesthetic clinic. Outcomes and costs were compared to national averages and reports from the literature.

**Methods:**

A retrospective chart review identified all adult injectable patients at the Resident Aesthetic Surgery Clinic at NYU Langone Health in 2021. Patient demographics, procedural data, complications, touch-ups, and surgeon fees were compiled. Factors predictive of return patronage were investigated as well. Data were analyzed and visualized in R.

**Results:**

In 2021, 223/233 consultations led to an injection (95.7% conversion rate) for 148 patients. Neurotoxin was most commonly injected into the upper face, while fillers were most commonly injected into the midface. Two complications from fillers (0.9% complication rate) and one touch-up (0.4% touch-up rate) were recorded. Surgeon fees were substantially less than national averages and comparable to other academic settings. Patients who received filler only at their first visit were significantly less likely to be return patrons in the same year.

**Conclusions:**

These data represent the largest and most detailed annual report of injectables from a plastic surgery resident aesthetic clinic, demonstrating high volume, low complication and revision rates, and reasonable costs to patients. Altogether, our study supports resident aesthetic clinics as a robust training modality.

## Introduction

Minimally-invasive procedures account for 85.2% of all aesthetic procedures in the United States.^1^ Of these minimally-invasive procedures, injectable neurotoxins (botulinum toxin; BoNTA) and fillers (hyaluronic acid; HA) account for 58.8%.^1^ Injectables are popular due to an array of benefits, including short procedural times, cost-effectiveness, and overall benign complication profile compared to surgical aesthetic procedures.^2^^,^^3^ With this surging popularity of injectables in recent years also comes rigorous competition for plastic surgeons. Nearly 70% of American Board of Cosmetic Surgery diplomats advertise themselves as “plastic surgeons” on social media, despite not being certified by the American Board of Plastic Surgery, creating a nebulous landscape for patients to navigate.^4^ Moreover, only about 40% of patients would choose a plastic surgeon to perform their injectable procedure.^5^

Although the market for minimally-invasive aesthetic procedures underwent a national decline from 2018 to 2020, a resurgence in injectables occurred in 2021.^1^^,^^6^^,^^7^ This surge in demand has been multifactorial, fueled in part by the COVID-19 pandemic and fixation on appearance through social media. Indeed, increased time and posts on social media has been associated with increased consideration of aesthetic surgery.^8^ Altogether, individuals have become more interested in minimally-invasive facial aesthetic procedures to enhance their appearance.^9^ With respect to demographics, the percentage of patients under 50 years old undergoing minimally-invasive procedures has reached its peak since 2015.^6^^,^^10^

Plastic surgery residents gain most of their aesthetic experience from resident-directed aesthetic clinics, in which senior residents evaluate patients independently and present a surgical plan to attendings.^11^, ^12^, ^13^ However, up to 10% of graduating residents in 2015 did not fulfill the injectable minimums set by the Accreditation Council for Graduate Medical Education (ACGME) in 2014,^14^^,^^15^ demonstrating substantial variability across residency programs. There continues to be a need to evaluate resident aesthetic clinics and their educational benefits. Prior studies of resident cosmetic clinics primarily focused exclusively on invasive surgical procedures, and there remains a dearth of literature regarding injectables in the academic setting. Herein, we report the largest and most detailed annual review of injectable (BoNTA and HA) procedures in a resident aesthetic clinic, focusing on sites of injection, complications, and costs. We also provide a model detailing the necessary components for other institutions interested in establishing their own resident-run clinics.

## Methods

### Aesthetic Surgery Clinic structure

We have previously described in detail the structure of the Resident Aesthetic Surgery Clinic in the Hansjörg Wyss Department of Plastic Surgery at NYU Langone Health.^12^ In brief, post-graduate year (PGY)-5 and PGY-6 residents rotate in the clinic for a total of 6 months, solely dedicating their time to the clinic. Patients are staffed with one of two dedicated attendings who provide feedback and supervision, helping residents refine their pre-procedure planning and follow-up skills. Once a patient establishes a patient-doctor relationship with a specific resident, that same resident will follow the patient throughout the remainder of their residency training. Residents have the flexibility amongst their other rotations to follow the patient longitudinally in the aesthetic clinic and continue to provide procedural and post-procedural care to their patient. Dedicated clinic front office staff streamline the process by assisting with procedure quotes and pre-procedural clearance. The resident aesthetic clinic does not engage in formal advertising, bills patients on a fee-for-service basis and offsets operational costs with revenue.

### Study design

We conducted a retrospective review of all injectable visits at the NYU Resident Aesthetic Surgery Clinic in 2021, tabulating demographic and procedural information from electronic medical records. Patients were excluded if outcome information was missing.

### Procedures and complications

Procedures were defined in a similar manner to ACGME resident case logs.^14^ Injectable procedures were counted by visit, but separated if a patient received both BoNTA and HA filler simultaneously. Bilateral facial areas injected were counted as one area. For example, a patient who presented to the clinic once for bilateral BoNTA injections to the orbicularus oculi and frontalis would be counted as one injectable visit with two facial areas injected. Complications were defined as adverse events distinct from typical side effects with injectables, such as temporary bruising, discoloration, or edema. Touch-ups were defined as a patient returning to the clinic shortly after an initial visit (typically a week later) for modulation/augmentation of the same prior procedure. Touch-ups were not billed, as these were an extension of the original visit.

### Data analysis

All data was analyzed in R and visualized using the ggplot2 package.^16^^,^^17^ A chi-squared test measured deviation in procedure frequencies across yearly quarters. Variables included in univariate analyses to predict return patronage included: age, gender, BMI, injectable type, past surgical history (PSH), procedure day of year, number of locations injected, and surgeon fee. Those with a *p*-value < 0.25 on univariate testing were purposefully selected for subsequent multivariate analysis. Variables were also assessed for collinearity and removed accordingly. A *p*-value < 0.05 was considered statistically significant.

## Results

### Patients receiving injectable are females in good general health

A total of 148 patients, 134 (90.5%) being female, were included (Table 1). Patients were in good health, with hypothyroidism and hypertension being the most common comorbidities at 8.8% and 7.4%, respectively. Active tobacco use was present in nine (6.1%) patients, while diabetes was present in two (1.4%) patients. The average age on date of injection was 48.4 ± 14.5 years old and average BMI was 23.4 ± 4.0 kg/m^2^.

**Table 1:** Demographics of injectable patients.

Patient demographics		*N* (%)
Gender	Female	134 (90.5)
	Male	14 (9.5)
Age	<30	16 (10.8)
	30–49	67 (45.3)
	50–69	50 (33.8)
	≥70	15 (10.1)
BMI^a^	<18.5	7 (4.7)
	18.5–24.9	74 (50.0)
	25–29.9	31 (20.9)
	30–39.9	10 (6.8)
Comorbidities	Hypothyroidism	13 (8.8)
	Hypertension	11 (7.4)
	Current tobacco use	9 (6.1)
	History of cancer	7 (4.7)
	Asthma	3 (2.0)
	Hyperlipidemia	3 (2.0)
	Anxiety	3 (2.0)
	Depression	3 (2.0)
	Diabetes	2 (1.4)
	Coronary artery disease	1 (0.7)

a Data unavailable for 26 patients.

### Neurotoxin injections are the most popular minimally-invasive procedure

**Figure 1:**
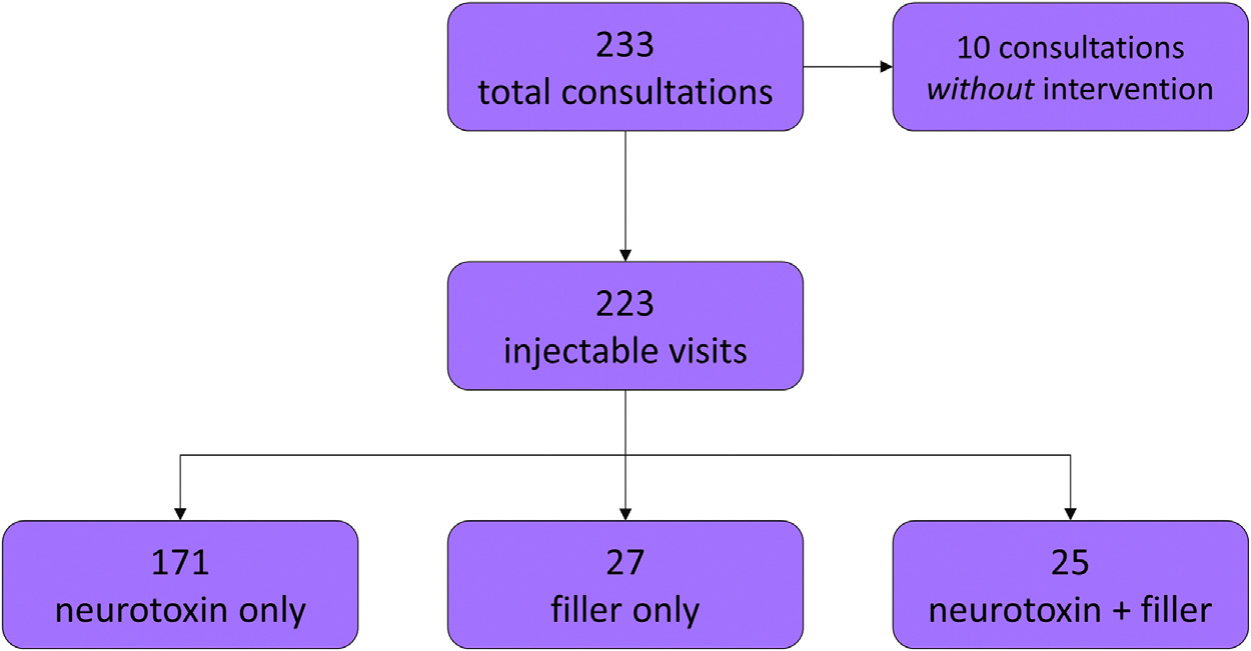
Flowchart of total annual consultations and injections. Breakdown of injectable consultations and interventions, including type of injectable received.

The resident aesthetic clinic produced 233 total consultations corresponding to 156 individual patients. We calculated a conversion rate of 95.7%, or 223 consultations resulting in an injectable procedure for 148 patients (Figure 1). The most popular injectable was BoNTA only (171 visits), followed by filler only (27 visits) and the combination of both BoNTA and filler (25 visits). Average volumes injected were approximately 37 units and 1 cc for BoNTA and HA, respectively.

**Figure 2:**
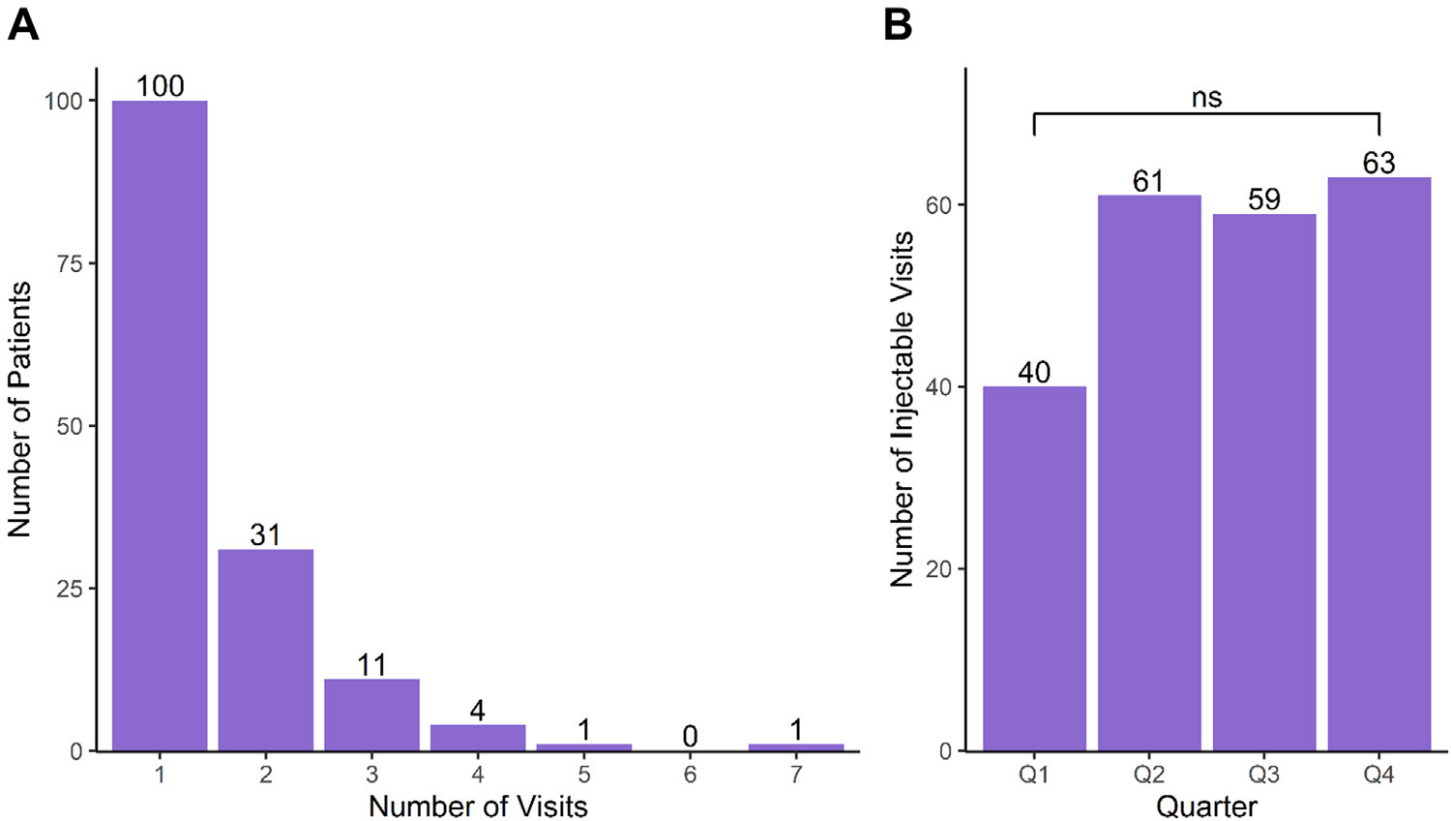
Frequency of aesthetic clinic injectable visits. Frequency of injectable visits by (A) number of annual visits and (B) yearly quarter.

Nearly all patients (95.1%) received an injection on the same day as their first consultation and over a third of patients returned to the clinic for another injection (Figure 2A). We did not observe any differences in the number of injectable visits by yearly quarter (*p* > 0.05; Figure 2B).

### Upper facial structures are most commonly injected

**Figure 3:**
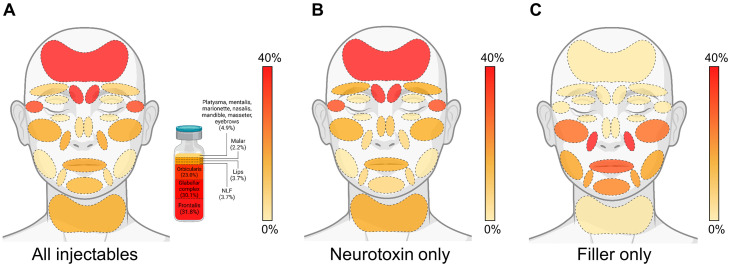
Common injectable locations. Anatomic heatmap of proportion of facial locations injected, split by (A) all injectables, (B) neurotoxin only, and (C) filler only.

Encompassing all injectable procedures, features of the upper face were most commonly injected, including the frontalis, glabellar complex, and orbicularis (Figure 3A). The nasolabial folds (NLF) and lips were the next most commonly injected areas. This trend was consistent for BoNTA injections (Figure 3B). However, HA was most commonly injected into the midface, including the NLF, lips, and malar region (Figure 3C).

### Injectables at the resident aesthetic clinic provide exceptionally safe, effective results

Of all 223 visits, we report two complications and one touch-up. Both complications occurred in patients who received filler. One patient experienced skin discoloration and the other experienced small nodule formation at the injection site, both of which resolved after hyaluronidase injections. One patient required touch-up of a BoNTA injection to the orbicularis. These instances correspond to a complication rate of 0.9% and touch-up rate of 0.4%.

### Injectables procedures are offered at reduced costs

We calculated an average surgeon fee of $389 for BoNTA injections and $283 for fillers (Table 2). This information, combined with average volumes injected, was used to calculate unit costs. We determined prices of $10.59/unit and $272/cc for BoNTA and HA, respectively. Overall, surgeon fees at the NYU Resident Aesthetic Clinic were less than national averages and comparable to other academic institutions (Table 3).

**Table 2:** Unit costs of injectables.

Injectable type		
Botulinum toxin	Avg units/visit	37
	Avg cost/unit	$10.59
	Avg cost/procedure	$389
Filler	Avg cc/visit	0.94
	Avg cost/cc	$272
	Avg cost/procedure	$283

**Table 3:** Cost comparison of injectables.

	NYU average surgeon fee	2021 Aesthetic Plastic Surgery (APS)	Δ%	2020 American Society of Plastic Surgeons (ASPS)	Δ%	Baylor 2018 (Wagner et al.^26^)	Δ%
Botulinum toxin	$389	$409	−5%	$466	−17%	$370	5%
Filler	$283	$766	−63%	$684	−59%	$0 (manufacturer donation)	

Average NYU surgeon fees for injectables compared to two national sources and another resident aesthetic clinic.

### Filler injections at first visit are associated with a lower likelihood of return patronage

Our final aim was to determine objective factors during a patient’s first injectable visit that were associated with return patronage within the same year. We controlled for procedure day of the year (ranging from 1 to 365), postulating that patients who first presented to the aesthetic clinic earlier in 2021 had a higher likelihood to return than those who first presented later in the year. Following variable selection using univariate analyses, our multivariate regression included the following predictors: age, injectable type, surgeon fee, and day of year. This model confirmed that patients first presenting to the clinic later in the year were significantly less likely to return by the end of the year. We also found that patients who received filler only at their first visit were significantly less likely to return in the same year (Table 4). No other objective factors were significantly associated with return patronage.

**Table 4:** Predictors of return patronage.

Category	Value	Odds ratio	95% CI
Injectable type	Filler only	**0.13**	**0.02–0.50**
	BoNTA & Filler	1.06	0.33–3.25
Procedure info	Day of year	**0.99**	**0.98–0.99**
Cost	Surgeon fee	1.00	1.00–1.00
Demographics	Age	1.02	0.99–1.05

Results of multivariate regression to determine factors associated with return patronage within the same year.

## Discussion

In this study, we present the experience of injectables at a plastic surgery resident aesthetic clinic, including details regarding injectable type, complications and touch-ups, costs, and factors associated with multiple visits, culminating in the largest and most extensive annual study to date. In total, the NYU Aesthetic Clinic provided care to 148 patients who presented for 223 injectable visits in 2021 (Figure 1). Patients receiving injectables were generally healthy females desiring BoNTA injections (Table 1, Figure 1), with nearly a third of patients returned to the aesthetic clinic within the same year (Figure 2A). BoNTA was frequently injected into features of the upper face, while fillers were commonly injected into parts the midface (Figure 3). We demonstrated exceptional safety and efficacy with only two complications that resolved with treatment and one touch-up. Compared to national averages, the cost of injectables to patients were far less at our resident clinic and were comparable to other institutional data (Table 3). Lastly, we determined that patients receiving filler were less likely to return to the aesthetic clinic within the same year, likely attributable to the longer duration of effect compared to neurotoxins (Table 4). In summary, we have demonstrated that our resident aesthetic clinic is a high-output training model for residents to gain experience with facial injectables under the expertise of attendings while providing care that is safe, effective, and affordable for patients.

Assessing the volume and diversity of resident aesthetic clinics’ experience with injectables is of interest for several reasons. First, it is necessary to evaluate resident training modalities, particularly in aesthetics where residents have historically struggled to gain adequate experience. Reasons for this are multifactorial; resident rotations in specific disciplines of plastic surgery have not been standardized by the ACGME, leading to substantial variability in the duration of rotations across programs.^18^ Second, aesthetic procedures are most often performed in private practices settings rather than academic institutions.^13^

The robust safety profile of injectables, coupled with rapid procedure times and results, is appealing for patients who wish to abstain from more invasive facial rejuvenation procedures. Of 196 BoNTA injections (either alone or in conjunction with fillers) in 2021, we documented no complications and one touch-up. One meta-analysis of complications from BoNTA injection to the upper face identified a complication rate of 16%, with common complications including headache/migraine, local skin reaction, and neuromuscular symptoms.^19^ The striking discrepancy between our study and this rigorous meta-analysis is likely explained by the lack of standard definition for a “complication”, along with the fact that many of our patients do not follow-up until their next injectable visit. The data on soft tissue fillers is not as concrete though; overall complication rates vary greatly and much of the literature data is rooted in self-reporting by patients, leading to bias and a general underreporting of complications.^20^, ^21^, ^22^, ^23^ One consistency is that the most common complication is nodule formation, accounting for 58.8% of all filler complications.^24^ However, the exact timeframe (early versus late onset) and classification of a nodule (versus granulomatous reaction, non-granulomatous reaction, sterile abscess, etc.) remains unstandardized in the literature.^25^ In our experience, we reported two complications (one nodule formation and one skin discoloration), both of which resolved with hyaluronidase treatment. Hyaluronidase is an effective treatment for filler complications, resolving nodules in 90.4% of cases.^23^ Compared to other academic institutional data, Wagner *et al.* recorded 153 injectable cases of BoNTA and/or filler in their aesthetic clinic in 2018, but did not document outcomes in their financial analysis.^26^ In contrast, the authors reported 23 BoNTA and/or filler injections in their resident aesthetic clinic over 13 years, resulting in no complications and one revision.^27^ Unfortunately, this small sample size and the paucity of literature data from resident aesthetic clinics makes it difficult to draw meaningful comparisons.

One comparison we can make though, in terms of clinic organization, is with the resident aesthetic clinic at the University of Wisconsin. Marks *et al.* recently highlighted the structure of their clinic, which is open Saturday mornings, organized by residents of all years, and is free to patients due to residency training product grants from by manufacturers.^28^ Although their clinic operates a fraction of the time as ours, they maximize educational yield for residents by selecting patients desiring multiple procedures, allowing residents to practice techniques efficiently. Their residents typically mirror injections with attendings when first learning, working towards clearly defined milestones each year, which is different than our institution’s clinic organized specifically by PGY-5/6 residents. Another difference in our clinic structure is that we ensure case volume by functioning 5 days per week, obviating the preference for patients desiring multiple procedures. Although Marks *et al.* highlight the benefit of product donations from manufacturers as a means to cut costs for these clinics, one downside is that this may not instill cost-conscientiousness in residents.^28^ While the authors outline their clinic structure and rationale quite well, they do not provide clinical outcomes data, which we build upon in our study.

As mentioned, cost is an important factor to consider not only in terms of patient accessibility, but also as a potential barrier to opening and maintaining a resident aesthetic clinic. Compared to national averages,^1^^,^^6^ we demonstrated that our average surgeon fees were slightly less for BoNTA and substantially less for fillers. However, the cost of injectable procedures is unique because it almost exclusively reflects the cost of the material itself, thereby limiting the discount that can be provided. That is, if a resident aesthetic clinic purchases materials from a supplier at market value, yet offers discounted rates to patients, the clinic will incur losses. Therefore, it is crucial to publish transparent data from existing resident aesthetic clinics in order to demonstrate financial viability and mitigate obstacles to opening future clinics.^26^ One possible solution is to obtain an industry sponsor that provides a certain allotment of materials at no cost, a strategy implemented by the resident aesthetic clinics at the University of Wisconsin^28^ and Baylor College of Medicine.^26^ Patients do not pay for injectables until the allotment is exhausted, in which patients will then pay full price. Baylor’s cost for BoNTA was $10/unit with an average of 36 units injected per visit,^26^ similar to our unit price of $10.59/unit and average of 37 units injected per visit.

Another unique aspect of injectables is the relatively short-term effects compared to surgical aesthetic procedures. As a result, patients tend to return for multiple injectable visits, naturally creating a longitudinal relationship between patient and physician. From both a profitability and resident education standpoint, it is useful to ascertain factors associated with return patronage. This gives physicians the ability to encourage patients to return by leveraging controllable factors such as cost. The ability to foster a larger, more consistent patient population enhances resident training and education. In our multivariate regression, we attempted to identify objective factors associated with return visits. Patients who received only filler at their first visit were less likely to return for additional injectables during the same year. This is intuitive, given the longer lasting effects of filler (12 months or longer) compared to BoNTA (3–4 months). Intriguingly, we were unable to identify any other objective factors predictive of return patronage from a patient’s first visit, suggesting that subjective factors may play a greater role. Although plastic surgery literature and education are typically focused on anatomical measurements and techniques to achieve optimal technical quality, patients often do not have the understanding to appreciate these nuances. Instead, patients typically infer the quality of their results through functional quality, which describes the way in which treatment is delivered and how patients perceive that treatment.^29^ That is, aspects of care such as empathy, a welcoming clinic environment, and communication are just as important as the success of the actual aesthetic treatment itself. In one study, injectable patients reported trust in their practitioner as the most important reason for return patronage, while cost was ranked the lowest.^29^ These findings can inform resident training strategies on how to maintain a loyal patient population. The LEAP framework (Listen, Educate/Empower, Align, and Perform) has been proposed to guide residents and fellows through cosmetic consults.^30^ This framework is built on quality communication with patients and includes patient education and enablement, leading to doctor-patient concordance and enhanced trust. LEAP and similar templates may be key to encourage return patronage in resident aesthetic clinics.

Some limitations of this study have been described above, including the lack of standardized definitions for complications, relatively short timeframe of the study, and its retrospective nature. Additionally, our regression analysis is imperfect; it is possible that a patient’s first visit of 2021 was not their first ever visit ever to the aesthetic clinic. Subsequent studies will aim to review a longer period to address this shortcoming. Another limitation is that we did not include patient reported outcome measures or assess resident perspectives on the utility of this training model for injectable procedures. Complication and revision rates are excellent objective proxies for the safety and efficacy of treatment. However, as recent literature has suggested, it is critical to subjectively assess patients’ trust with providers and satisfaction with their outcome. Equally important is gauging residents’ confidence with performing injectables, which we recommend in future studies to more holistically evaluate the educational benefit of resident aesthetic clinics.

## Conclusions

The surging demand for minimally-invasive aesthetic procedures, particularly facial injectables, necessitates evaluation of plastic surgery residency training modalities. This study demonstrates our experience with facial injectables, namely BoNTA and HA fillers, at the Resident Aesthetic Surgery Clinic at NYU Langone Health. We report the highest annual number of injectable procedures to date, while maintaining a low complication rate, revision rate, and reasonable cost to patients. We also provide transparent financial data on these procedures, allowing other institutions to estimate financial viability prior to integrating a similar model into their training programs. Through our analysis and collaborative efforts from attendings, administration, and clinical staff, we establish that resident aesthetic surgery clinics provide excellent experience in injectable facial aesthetic procedures to residents while maintaining safe and reliable treatment for patients.

## Funding

The authors declare no funding support was provided regarding the publication of this article.

## Ethical approval

This study adhered to protocols approved by the Institutional Review Board (IRB), in accordance with the Code of Federal Regulations on the Protection of Human Subjects (45 CFR Part 46) and Declaration of Helsinki. Written consent was provided, by which the patients agreed to the use and analysis of their data.

## Declaration of competing interest

The authors declare no conflicts of interest regarding the publication of this article.
